# The Home and Infirmary for Sick Children, Sydenham

**Published:** 1912-05-04

**Authors:** 


### THE HOME AND INFIRMARY FOR SICK CHILDREN, SYDENHAM.

This suburban institution is housed in a large converted mansion of mid-Victorian appearance in the Sydenham Road, Lower Sydenham. As a specimen of contemporary architecture it was no doubt creditable, and suitable for its original purpose; but it is very ill-adapted for conversion into a hospital. Short of rebuilding the whole fabric it is hardly possible to satisfy the most modern and up-to-date requirements of what a children's hospital should be, and for such an extensive programme there are, unfortunately, no funds available nor any visible prospect of obtaining them. It must be understood, therefore, that in mentioning some of the drawbacks and defects of the buildings there is no censure implied upon the Committee, who have had to make the best of the accommodation at their disposal and to contrive ways and means of adaptation out of a small income.

### The Wards.

The main building is on three floors. The ground floor is devoted chiefly to the matron's room, nurses' sitting-room, committee-room, etc., but there is also one ward for eight cots. On the first floor are surgical wards, on the second floor medical wards. There are also an operating theatre, to which reference will be made presently, and a children's playroom. A recent addition to the building is a series of verandas to the various floors which allow of the bedridden children being taken out into the open air when climatic conditions are suitable.

The wards are, of course, simply converted dwelling-rooms, and in consequence they are not provided with sufficient window space for their present purpose. The medical wards are especially dark and gloomy; in fact, condemnation from this point of view would have to be expressed very strongly were it not for the difficulty of suggesting any feasible remedy. The surgical wards are not so defective in this respect, but even they are very far from being properly lighted. The wards are warmed by open fires, efficiently screened by large fenders so that the children cannot suffer accident. Thus the ventilation is that of the mansion as originally constructed.

### Where Patients Come From.

The cots are of a good pattern and are well kept. The patients, for whom a small payment is charged, are drawn partly from the neighbourhood and from the out-patient department, and partly from some of the London hospitals, whence chronic or convalescent cases are sent to Sydenham for continuation of treatment or for more complete recovery. Queen Alexandra's Hospital and the Great Northern Hospital, in particular, take advantage of the accommodation thus provided; but cases are accepted from other hospitals also. The Home and Infirmary does not, however, merely undertake the after-care of sick children; cases from the neighbourhood are accepted and treated from start to finish, and operative surgery is a recognised part of the amenities of the institution. The Whittuck Home at Broadstairs, with accommodation for twelve children, is a special feature of the Sydenham Home and Infirmary.

There are 57 cots in the home, almost all of which were occupied at the time of our inspection. The children are mostly from two to seven years of age, though there is a small ward for infants and a few older children are also warded. The children look clean, bright, and happy, and are obviously well cared for and well fed. A fair proportion of them are confined in surgical jackets, splints, and other appliances for the relief of spine and joint diseases.

### Drawbacks and Disadvantages.

The wards themselves, as has been intimated, are far from ideal. The floors are covered with linoleum, which is in good condition; probably underneath are the ordinary deal boards as originally put in by the builder, and no doubt also a collection of dirt and miscellaneous dust. Short of relaying the floors, the plain linoleum is probably the most practicable way of dealing with this problem, though it is an unsatisfactory expedient borne of necessity. The ward sinks, closets, bath-rooms, and kitchens are entered direct from the wards; this arrangement is, from a sanitary standpoint, indefensible, but again it is not easy to suggest any other remedy than complete demolition and reconstruction. Considering the small window-area and the other disadvantages of the wards, it would probably be better to reduce by 15 or 20 per cent. the number of cots in each ward, for though the wards are not so crowded as to be unworkable, still the children would fare better if each had a larger average cubic space of air.

The operating theatre is small and has only a side light from a window; the sterilisers are in the theatre. In a small ante-room dressings are done, and the anæsthetist has to share possession of it with a number of antiseptic stores of various kinds. This ante-room would appear to provide the normal entrance to the theatre, a state of things which prevails in many institutions where there is less excuse for it than in the present case. Inconvenient as this theatre must be, judged by up-to-date standards, there is yet no reason why good work should not be, and doubtless is, done in it. Like the rest of the Home, it is a makeshift expedient, but it serves a very useful purpose.

### Nursing Deficiency.

The nurses' sitting-room is a cheerful and comfortably furnished room, and the matron's apartments are also satisfactory. The matron, Miss Ray, from whom we received every possible facility and courtesy, is assisted by two trained sisters, and there are also ten probationers, who are trained for their duties by the three seniors. On the occasion of our visit three nurses were eating a meal in one of the ward kitchens, and we saw no nurses' dining-room, an omission which no deficiency of means or space can be allowed to excuse.

### The Extra Block.

At the back of the main building, and separated from it by a space of perhaps 20 feet, is a smaller block which serves several purposes. The ground floor is the out-patient department. This was not originally part of the scheme, but was first introduced in order that the staff might supervise the progress of discharged patients. From that it has grown until at present there are sometimes fifty or sixty patients in a day.

The accommodation for this considerable clinic is meagre in the extreme. The small waiting-room is capable of holding perhaps half the figure last mentioned of mothers and children, and we learn that in fact on busy days patients have actually to wait outside.

One curious piece of furniture is a horizontal bar hung on ropes from the ceiling in the waiting-room. If this is required for straightening out (for diagnostic purposes) backs which are deformed from muscular weakness, it should surely be in the doctor's room; if it is for recreative purposes a room full of sick children is hardly the best place for it. As we shall have some remarks to make presently about the out-patient department, it will be sufficient to state here that the accommodation provided is defective in the extreme, and that there is less excuse than with regard to the main building, for it would be a simple matter to make the necessary extensions.

### The Isolation Floor.

The first floor of this same block is an isolation ward for cases of infectious disease occurring among the in-patients, with a room for a nurse, kitchen and other offices. Access to this floor is obtained only from the main building by means of a connecting iron way, and the isolation ward is thus cut off from the floors above and below. The top floor is devoted to sleeping accommodation for the nurses, and is connected by a covered-in passage way with the corresponding floor of the main building. The beds are placed in cubicles, and the furniture is ramshackle and comfortless. The lighting and ventilation of these cubicles are not at all what they ought to be; this is a matter which stands in urgent need of amendment.

The grounds in which these buildings (and a separate mortuary) stand are not large; probably the present policy of abandoning the attempt to grow produce is a wise one, and certainly the recent provision of a sandpit for the children to play in during the summer months is an excellent scheme.

### Finance and Policy.

At the present time, it would seem, the finances of this Home are not in a very flourishing condition. How far this is a result of a lack of interest among the inhabitants of Sydenham, and how far of too ambitious a programme on the part of the management, is difficult to say. There are, however, certain reflections which a visit to this Home evokes.

In the first place it is obvious that the available accommodation is insufficient for the purpose of treating 57 children. The out-patient department and the arrangements for the nursing staff prove this.

A reduction in the number of cots to, let us say, 35 or 40, would enable better provision to be made for the nurses and would at the same time relieve the Committee of a considerable annual charge for maintenance. At present the staff of trained nurses is not large enough to deal with both the patients and the training of the probationers. It is evident that either there is no trained nurse on night duty, or else that one of them has to manage both medical and surgical wards during the daytime, with possibly the theatre thrown in on operating days.

### A Mistaken Department.

Secondly, the out-patient department on its present scale is a mistake; for one reason because it has been allowed vastly to outgrow the accommodation, and for another because the primary object of the charity is clearly to act as an auxiliary agency to recognised hospitals. If it is to be part of the considered policy of the managers to make this a *hospital* for local needs, rather than a *home and infirmary*, then it behoves them to act energetically in raising funds to rebuild and properly equip their institution.

But if not, their chief endeavour should be to promote the efficiency of the Home along its proper lines. The impression gained by our inspection is that the Committee are attempting more than they have the support necessary to carry out, perhaps under pressure from an ambitious medical staff. If that is the case, they must either strengthen their position by stimulating local interest and by working hard in collecting subscriptions, or else they must refuse to allow the staff to commit the Home to a policy and a programme for which financial resources are not available. We do not presume to advise which of these policies should be followed; the local conditions must be taken fully into consideration and a decision taken accordingly.

### Essential Reforms.

Lastly, there are certain details, as have been indicated, which require amelioration without delay. The nurses' cubicles, for one thing; the out-patient department, for another; and the fortification of the nursing staff by a larger percentage of fully-trained women for a third. When these have been taken in hand the Sydenham Home will, despite its disadvantages structurally, do good work in the future as it has done in the past.

